# Fulminant respiratory failure due to severe pneumothorax after re-do coronary artery bypass grafting treated with veno-venous extracorporeal membrane oxygenation

**DOI:** 10.1093/jscr/rjae360

**Published:** 2024-05-29

**Authors:** Akito Inoue, Ryohei Ushioda, Kazuki Miyatani, Kentaro Shirakura, Nobuhiro Mochizuki, Hideki Isa, Yuki Setogawa, Masahiko Narita, Fumitaka Suzuki, Aina Hirofuji, Ryo Okubo, Shingo Kunioka, Masahiro Tsutsui, Kamiya Hiroyuki

**Affiliations:** Department of Cardiac Surgery, Asahikawa Medical University, Asahikawa, Midorigaoka 1-1-1, Japan; Department of Cardiac Surgery, Asahikawa Medical University, Asahikawa, Midorigaoka 1-1-1, Japan; Department of Cardiac Surgery, Asahikawa Medical University, Asahikawa, Midorigaoka 1-1-1, Japan; Department of Cardiac Surgery, Asahikawa Medical University, Asahikawa, Midorigaoka 1-1-1, Japan; Department of Cardiac Surgery, Asahikawa Medical University, Asahikawa, Midorigaoka 1-1-1, Japan; Department of Cardiac Surgery, Asahikawa Medical University, Asahikawa, Midorigaoka 1-1-1, Japan; Department of Cardiac Surgery, Asahikawa Medical University, Asahikawa, Midorigaoka 1-1-1, Japan; Department of Cardiac Surgery, Asahikawa Medical University, Asahikawa, Midorigaoka 1-1-1, Japan; Department of Cardiac Surgery, Asahikawa Medical University, Asahikawa, Midorigaoka 1-1-1, Japan; Department of Cardiac Surgery, Asahikawa Medical University, Asahikawa, Midorigaoka 1-1-1, Japan; Department of Cardiac Surgery, Asahikawa Medical University, Asahikawa, Midorigaoka 1-1-1, Japan; Department of Cardiac Surgery, Asahikawa Medical University, Asahikawa, Midorigaoka 1-1-1, Japan; Department of Cardiac Surgery, Asahikawa Medical University, Asahikawa, Midorigaoka 1-1-1, Japan; Department of Cardiac Surgery, Asahikawa Medical University, Asahikawa, Midorigaoka 1-1-1, Japan

**Keywords:** extracorporeal membrane oxygenation, post-operative rehabilitation, pneumothorax

## Abstract

This case report details the management of a 79-year-old man who developed massive postoperative pneumothorax following redo coronary artery bypass grafting due to severe lung adhesions. We successfully treated the patient using veno-venous extracorporeal membrane oxygenation without femoral cannulation, allowing for early rehabilitation initiation. Veno-venous extracorporeal membrane oxygenation is a reasonable option for cases of severe respiratory failure due to pneumothorax with lung destruction caused by re-sternotomy during re-do cardiac surgery.

## Introduction

In re-do cardiac surgery through re-sternotomy, the lungs are often severely adhered to the sternum, resulting in postoperative pneumothorax. In most cases, it can be treated by conservative management with lowering positive end-expiratory pressure (PEEP) and leaving chest drainage tubes, but higher PEEP needed in patients with impaired oxygenation due to pulmonary congestion. Here we report a patient treated with veno-venous extracorporeal membrane oxygenation (VV-ECMO) for massive postoperative pneumothorax after re-do coronary artery bypass grafting (CABG).

## Case report

A 79 years old man who underwent CABG 10 years ago was brought to his previous physician with a chief complaint of chest pain and was sent to our hospital for CABG after being diagnosed with acute coronary syndrome. At the previous hospital, he underwent coronary angiography and revealed that his left main coronary artery and all bypass grafts were occluded. A computed tomography (CT) scan performed at our hospital showed bilateral pulmonary congestion and pleural effusion, as well as prominent emphysema. We performed on-pump beating CABG with a saphenous vein graft (SVG) from the aorta to the left anterior descending artery and another SVG as a T-composite graft to the obtuse marginal artery. Both lungs were severely adhered to the chest wall due to previous CABG and therefore injured at the time of chest opening. As air leaks were observed immediately after surgery, but oxygenation was maintained, the patient was admitted to the intensive care unit.

**Figure 1:**
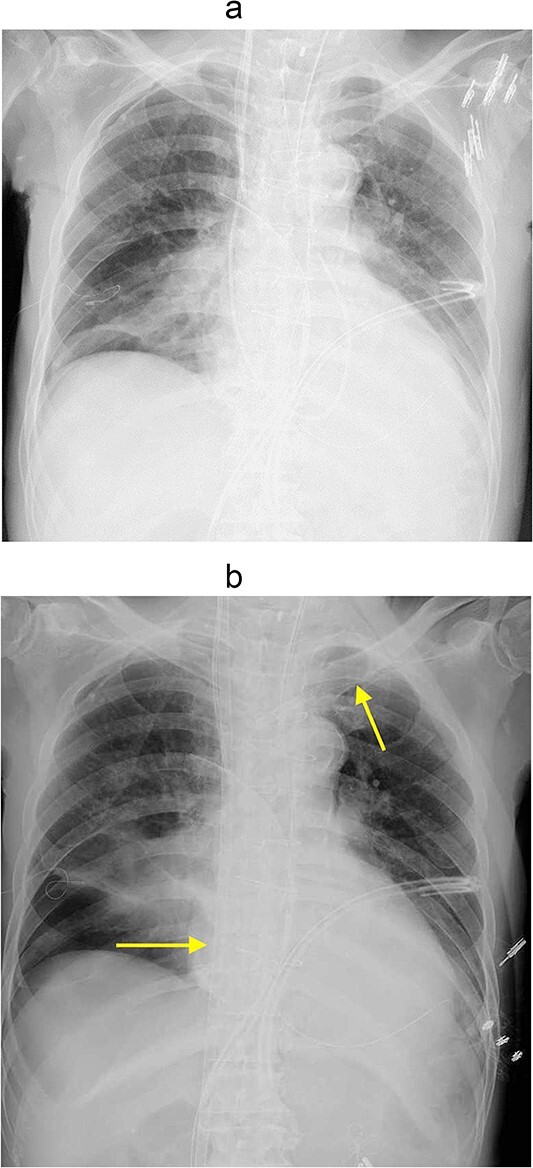
(a) Chest Xp immediately after the operation. (b) Chest Xp after VV-ECMO (arrow: venous cannulations).

**Figure 2:**
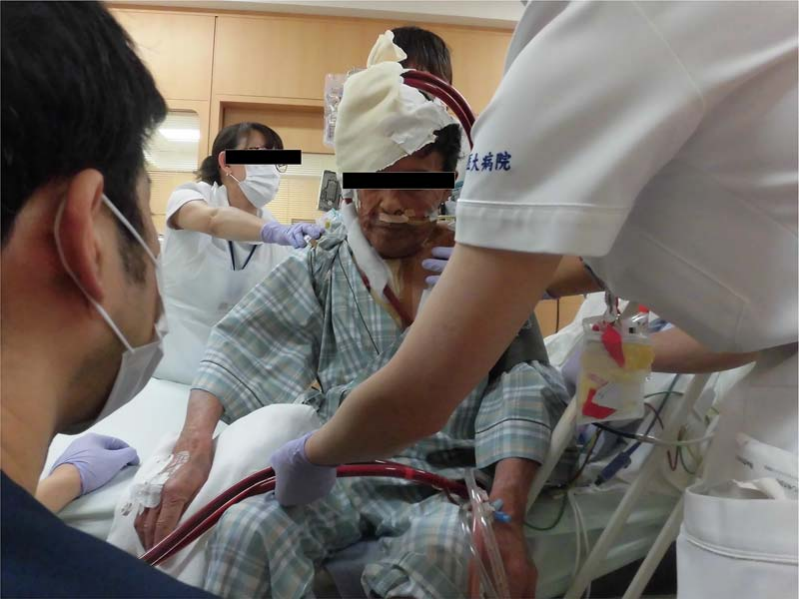
Photograph of the patient with VV-ECMO without intubation.

However, 3 hours after entering the intensive care unit (ICU), PaO_2_/FiO_2_ ratio was severely reduced to the 70s (Fig. 1a). It was necessary to apply high PEEP for cardiogenic shock with pulmonary edema, but the higher the PEEP, the more air leakage there was. Although hemodynamics was stable, his respiratory status was poor and reoperation was deemed difficult. Therefore, VV-ECMO was atarted on postoperative day (POD) 2. To facilitation mobilization and rehabilitation to improve lung oxygenation, cannulas were not inserted through the groin; a drainage cannula was inserted via the right internal jugular vein to the vena cava inferior and oxygenated blood was perfused into the left subclavian vein (Fig. 1b). On POD3, oxygenation has improved and the patient was extubated. He was managed with VV-ECMO inserted, as air leak was still allowed. On the same day, rehabilitation intervention was also started (Fig. 2). The air leak disappeared on POD4, and the VV-ECMO was removed on POD6. He was discharged from our hospital on POD13, and 6 years after the surgery, he is still alive and well.

## Discussion

In re-do cardiac surgery, lung injury happens sometimes due to severe adhesions between the lung and the chest wall. Generally, continuous thoracic drainage is performed for observation, and if air leak persists, pleurodesis or surgical intervention is performed to improve the situation. In the present case, however, the patient was in a dilemma: higher PEEP was required for congestive heart failure with lung edema, but higher PEEP increased air leak and worsened his respiratory condition, making ventilatory management difficult. Veno-arterial extracorporeal membrane oxygenation (ECMO) may be an option, but it does not work if a patient has very poor pulmonary function and preserved cardiac output, as in the present case, due to problematic mixing zone of oxygenized and un-oxygenized blood [1]. Therefore, to improve oxygenation situation and to reduce PEEP at the same time, VV-ECMO was introduced in the patient. To the best of our knowledge, this is the first case in which VV-ECMO has been used for pneumothorax after open heart surgery.

According to ELSO guidelines, ECMO is indicated when there is a significant decrease in P/F ratio or a severe air leak [2]. Wu *et al.* [3] reported a case of pneumothorax in a patient with pneumocystis pneumonia who developed pneumothorax on a ventilator. Kawaguchi *et al.* [4] also noted that VV ECMO is a good indication for patients with severe air leaks, as they successfully saved the life of a patient with severe pneumonia who developed a refractory pneumothorax while on a ventilator. The use of ECMO improves oxygenation and, in addition, allows the lungs to rest because PEEP can be lowered. In the present case, the patient was even extubated to avoid positive airway pressure during VV ECMO therapy, resulting in rapid spontaneous healing of pneumothorax.

Another advantage of VV ECMO is that walking and simple rehabilitation can be performed with the device in place. In fact, Abrams *et al*. [5] reported successful early rehabilitation of 35 patients under ECMO insertion. In the present case, femoral cannulation was avoided and rehabilitation could be started very early, resulting in rapid improvement of general conditions.
